# Altitudinal and household breeding patterns of the medically important mosquitoes *Aedes aegypti, Aedes albopictus* and *Culex quinquefasciatus* in Nepal

**DOI:** 10.1371/journal.pone.0345285

**Published:** 2026-03-19

**Authors:** Sunita Baral, Pramod Joshi, Bishnu P. Marasini, Ishan Gautam, Meghnath Dhimal, Ruth Muller

**Affiliations:** 1 Nepal Health Research Council, Kathmandu, Nepal; 2 Natural History Museum, Tribhuvan University, Swyambhu, Kathmandu, Nepal; 3 Institute of Tropical Medicine, Antwerp, Belgium; World Health Organization, Regional Office for South-East Asia, INDIA

## Abstract

Medically relevant mosquitoes have established populations across Nepal, adapting to diverse environmental conditions and altitudes. In addition to arbovirus vectors, the presence of *Culex quinquefasciatus*, a primary vector of lymphatic filariasis, underscores the need for comprehensive mosquito surveillance. This study investigated the presence, abundance, container preferences, and altitudinal distribution of *Aedes aegypti*, *Aedes albopictus*, and *Cx. quinquefasciatus* across selected districts in Nepal. We also examined the co-occurrence of *Aedes* species and their association with container type and location. An entomological survey was conducted along an altitudinal transect from 140 meters above sea level (m asl) (Chitwan) to 2340 m asl (Dolakha). Immatures were collected from 1,157 water-holding containers across 636 households using dipper and dropper techniques, while adults were captured using BG-Sentinel traps. Immatures were reared to adulthood for identification, while non-reared specimens were identified via microscopic slides. A total of 1,323 immature and 158 adult mosquitoes were recorded. Among immatures, *Ae. albopictus* (47.6%) was the most abundant, followed by *Ae. aegypti* (43.4%). Adults were predominantly *Cx. quinquefasciatus* (82.9%). Plastic bottles and drums were the most common larval habitats. *Aedes* species were found across the study transect, from 140 to 2066 m asl. The altitude did not have a statistically significant effect on the number of collected mosquitoes (*p* = 0.563). These findings highlight the widespread presence of *Aedes* and *Culex* species across different altitudes and breeding habitats. The predominance and co-occurrence of dengue vectors and *Cx. quinquefasciatus* in nearby households underscore the need for integrated vector surveillance and control strategies in Nepal.

## Introduction

Vector-borne diseases accounted for more than 80% of global population risk, with mosquito-borne diseases (MBDs) being the major contributor [1]. MBDs are considered a major health threat in Southeast Asian countries, including Nepal. They account for more than 700 million infections with 1 million deaths worldwide [2]. Nepal is now endemic to various MBDs such as malaria, lymphatic filariasis, Japanese encephalitis, chikungunya and dengue posing a significant public health burden [3]. Though the first case of dengue in Nepal was reported in a Japanese volunteer in 2004 [4], the very first outbreak in Nepal and the detection of the primary dengue vector *Ae. aegypti* in low-altitude regions of Terai bordering India were confirmed in 2006 [5]. Since then, sporadic dengue cases and dengue outbreaks have occurred from tropical lowlands to subtropical hilly regions year by year, with the so far largest dengue outbreak affecting all districts previously considered as non-endemic in 2022 [6–8]. Chikungunya virus (CHIKV) was first reported in Nepal in 2013, and subsequent studies confirmed its presence in different areas, including the Terai regions during the 2014–2015 outbreak [9,10].

*Aedes aegypti* and *Ae. albopictus* are not only the primary and secondary vectors capable of transmitting the dengue virus [11], but are also the principal vector species capable of transmitting the CHIKV [12]. In the case of Nepal, *Ae. albopictus* was reported in the 1950s [13] and *Ae. aegypti* in 2006 [14]. Genomic data indicate a non-gradual expansion of *Ae. aegypti* in Central Nepal [15]. Now both vectors are recorded at altitudes up to 2438 m above sea level [16] and *Ae. albopictus* was reported up to 2520 m [17]. Between the 1950s and the detection of *Ae. aegypti* in Kathmandu in 2009, immature and adult stages of DENV and CHIKV vectors were recorded in the lowland and middle mountain regions [14,18,19]. However, the expansion of these vectors to the high mountainous regions up to 2438m asl [16,20], highlights the increasing adaptability of these vectors to higher altitudes and the growing risk of vector-borne diseases in previously unaffected areas. This is attributed to the fact that the warming in higher mountain regions is more prominent than in the lowland areas such as Terai and Siwalik [21–23].

Besides dengue vectors, other vectors have already established their distribution in Nepal. Lymphatic filariasis (LF), a mosquito-borne parasitic disease, is transmitted by the bite of different species of mosquitoes (*Culex, Anopheles* & *Aedes*) and is caused by thread like filarial worms; *Wuchereria bancrofti* and *Brugia* species. In Nepal, *Culex quinquefasciatus* is the principal vector of LF. Sixty-three out of 77 districts of Nepal are considered endemic to LF on the basis of Immunochromatography Test (ICT) card surveys, morbidity reporting, vector density, sanitation status of the districts, and geo-ecological comparability [24]. In the absence of a vaccine and effective treatment for several of these diseases, symptomatic treatment and vector control programs are at present the only possible approaches for tackling these infections.

The principal vector of LF, *Cx. quinquefasciatus*, has been recorded at elevations up to 2100 meters above mean sea level (m asl) in central Nepal [20]. Similarly, *Cx. tritaeniorhynchus*, the principal vector of Japanese encephalitis in Nepal, has been observed at elevations up to 2000 m asl in Eastern Nepal [25]. Additionally, malaria vectors such as *Anopheles fluviatilis*, *Anopheles annularis*, and the *Anopheles maculatus* complex have been recorded in eastern Nepal up to 1820 m asl, with their larvae found at elevations as high as 2310 m asl [25]. Similarly, *Aedes japonicus*, *Anopheles farauti,* and *Culex sasai* were reported for the first time from Nepal from various landscapes [26]. However, their function as vectors in the spread of human pathogens in the area is still unknown and needs more research.

Our study aimed to examine the ecological pattern of three medically important mosquito species at varying elevations: *Ae. aegypti*, *Ae. albopictus,* and *Cx. quinquifasciatus*. In particular, we addressed the following research questions: 1. What is the relative abundance of *Ae. aegypti*, *Ae. albopictus*, and *Cx. quinquefasciatus* across different altitudes in Nepal? 2. Which container types are most frequently used as breeding habitats for *Aedes* mosquitoes? 3. How does altitude influence the distribution of *Ae. aegypti*, *Ae. albopictus*, and *Cx. quinquefasciatus*? This study also aimed to record the presence of other mosquito vectors found during the collection process to gain a more comprehensive understanding of the overall vector ecology in the area.

## Methods

### Study design

The sampling was carried out during the post-monsoon season from 30^th^ October 2021–30^th^ November 2021. Three districts (Chitwan, Kaski, and Dolakha) were selected purposely based on the highest number of dengue cases determined by the reports published by EDCD (Epidemiology and Disease Control Division) Nepal in 2019 and also in order to reflect various altitudinal and ecological settings. Altitude and temperature were chosen as the main environmental factors affecting vector ecology in order to lessen the influence of covariates like direct road connectivity, wind corridors, and urbanization. Within three districts, 35% of the governmental health facilities were randomly selected by generating a random number generator in Microsoft Excel resulting in 15 health facilities from Chitwan, 18 health facilities from Kaski, and 20 health facilities from Dolakha districts, respectively. From within those health facilities, 636 households (12 per health facility) were selected conveniently along an altitudinal gradient ranging from lowland to highland, from 140 m asl (Chitwan) to 2339 m asl (Dolakha).

The elevation of collection sites in Chitwan district ranged from 140 m to 1066 m asl, with temperatures ranging from 23°C to 30°C with minimal rainfall. The population density is approximately 325 people per km². The collection sites in Kaski were located from 680 m to 1957 m asl and average temperature ranges from 10 °C to 20 °C. The collection month is among the driest months, with minimal to no rainfall. The population density is approximately 297 people per km². The elevation of sampling sites in Dolakha ranges from 977 m to 2339 m asl. The district’s annual average temperature is approximately 31.06 °C. The population density is about 79 people per km².

### Entomological survey

In the study areas, all types of possible breeding habitats, like cemented tanks, plastic drums, metal drums, mud pots, discarded tyres etc., were searched in and around the houses for *Aedes* immatures. A total of 180 households from Chitwan, 240 from Dolakha, and 216 from Kaski were chosen. The immature collections were done using the dropper and dipper technique, whereas adults were collected using the BG-Sentinel traps equipped with a BG-Lure (Biogents AG, Regensburg, Germany) without carbon dioxide for 24 hours. BG-Sentinel traps were placed in the residential areas for safety purposes. The collected larval and adult mosquito samples were transported to the laboratory of the Natural History Museum, Tribhuvan University, Swayambhu, Kathmandu, Nepal. Larvae and pupae were reared to adulthood in plastic cups containing water from breeding habitats at room temperature covered with a net for adult emergence. Emerged adults were morphologically identified using the standard identification key by Darsie and Pradhan [13]. Permanent slides of larvae unable to emerge were made and identified using a stereomicroscope based on larval characteristics.

### Data analysis

Data obtained was entered into the Microsoft Excel spreadsheet and analysed using SPSS version 23 and R version 4.3.3. Binary logistic regression analysis was used to assess the associations between the presence of *Ae. aegypti*, *Ae. albopictus* and co-occurrence with container type, location (indoor/outdoor) and district. However, due to sparse data and instability of model estimates across some containers categories, multivariate logistic regression analysis could not be performed. The statistical significance of associations was evaluated using p-values, with a threshold of p < 0.05 considered significant. In accordance with World Health Organization Southeast Asia Regional Office (WHO SEARO) guidelines [27], following entomological indices have been calculated: House Index (HI, percentage of houses positive for *Aedes* larvae), Container Index (CI, percentage of containers positive for *Aedes* larvae), and Breteau Index (BI, number of containers positive for *Aedes* larvae per 100 houses). The container preference of *Aedes* larval breeding was additionally assessed by calculation of the breeding preference ratio (BPR = % of positive containers/% of wet containers) [16]. The mean ratio between *Ae. aegypti* and *Ae. albopictus* was calculated as the percentage of *Ae. aegypti* positive containers divided by the percentage of *Ae. albopictus* positive containers for each container type. Similarly, the effect of elevation on mosquito abundance was evaluated using a generalized linear model (GLM) with a Poisson distribution.

### Ethical statement

Ethical clearance for the entomological survey was obtained from the Ethical Review Board of Nepal Health Research Council under the Research Proposal ID: 206/2021. Since the study did not involve human participants, informed consent was not required. However, permission from household members was granted prior to sampling.

## Results

### Mosquito distribution and abundance across study sites

A total of 1,157 (Chitwan = 197, Kaski = 644 and Dolakha = 316) water-holding containers from 636 households were surveyed, yielding 1,323 immature mosquitoes (Chitwan = 311, Kaski = 467 Dolakha = 545). Additionally, 158 (Chitwan = 121, Kaski = 28, Dolakha = 9) adult mosquitoes were collected from both inside and around the houses. Both dengue vectors, *Ae. aegypti* and *Ae. albopictus*, as well as the lymphatic filariasis vector, *Cx. quinquefasciatus*, were recorded throughout the study transect. Other species showed more restricted distributions, with *Armigeres* spp. recorded up to 1,912 m asl, *Aedes indicus* up to 1,657 m asl, *Pallidus triatus* up to 1,555 m asl, *Anopheles subpictus* up to 1,081 m asl, and *Aedes subalbopictus* up to 591 m asl.

**Fig 1:**
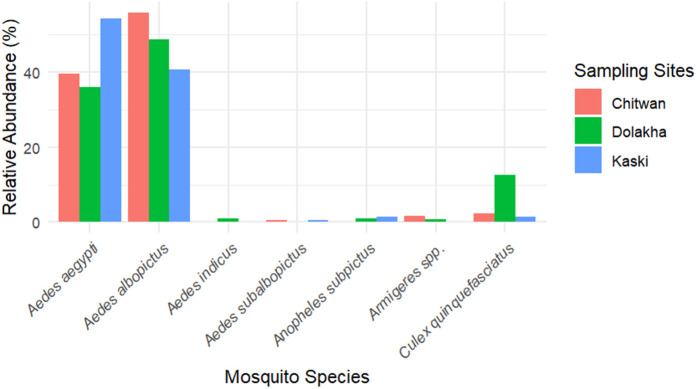
The relative abundance of mosquito immatures across Chitwan, Kaski, and Dolakha.

Among the immatures, 91% are known as dengue vector species (*Ae. aegypti*: 43.4%, *Ae. albopictus*: 47.6%). Other immature mosquitoes belong to *Cx. quinquefasciatus* (6.1%), *Anopheles subpictus* (0.8%), *Armigeres* spp. (0.7%), *Ae. subalbopictus* (0.4%) and *Ae. indicus* (0.4%). Immature *Ae. aegypti*, *Ae. albopictus*, and *Cx. quinquefasciatus* were recorded from all study areas (Fig 1), indicating their widespread presence and adaptability to varying environmental conditions. Notably, all three species were observed at altitudes as high as 2067 m, highlighting their ability to thrive across a broad altitudinal range, including high-altitude environments. In contrast, *Ae. subalbopictus* was recorded exclusively in Chitwan and Kaski, *Armigeres* spp. in Chitwan and Dolakha, *Anopheles subpictus* in Kaski and Dolakha and *Ae. indicus* in Dolakha. The relative abundance of immature mosquitoes varies between three districts. The relative abundance of immature *Ae. albopictus* was found to be highest in the lowlands (Chitwan: 55.9%) whereas immature *Ae. aegypti* were found to be highest in the hilly region (Kaski: 54.4%). Likewise, the relative abundance of *Cx. quinquefasciatus* was found to be highest in Dolakha (12.5%). House index (HI) for *Ae. albopictus* (16.67) and *Ae. aegypti* (15.42) were found to be highest in Dolakha whereas container index (CI) and Breteau index (BI) for both *Ae. albopictus* (CI: 9.14; BI: 10) and *Ae. aegypti* (CI: 9.64; BI: 10.56) were found to be highest in Chitwan (Table 1).

**Table 1:** Prevalence of *Ae. aegypti* and *Ae. albopictus* larvae across different districts in Nepal.

*Aedes aegypti* larvae								
District	No. of house visited	% found positive	No. of wet containers searched	% found positive	No. of larvae	HI (%)	CI (%)	BI (%)
**Dolakha**	240	15.41	316	7.91	118	15.42	7.91	10.42
**Kaski**	216	1.48	644	2.17	144	14.81	2.17	6.48
**Chitwan**	180	10.55	197	9.64	66	10.56	9.64	10.56
***Aedes albopictus* larvae**								
**District**	**No. of house visited**	**% found positive**	**No. of wet containers searched**	**% found positive**	**No. of larvae**	**HI (%)**	**CI (%)**	**BI (%)**
**Dolakha**	240	16.66	316	7.27	180	16.67	7.28	9.58
**Kaski**	216	14.35	644	3.10	129	14.35	3.11	9.26
**Chitwan**	180	12.77	197	9.13	132	12.78	9.14	10.00

Summary of house index (HI), container index (CI), and Breteau index (BI) for *Ae. aegypti* and *Ae. albopictus* larvae in Chitwan, Kaski, and Dolakha districts.

Among the adults, the majority of the species belong to the principal vector of lymphatic filariasis, *Cx. quinquefasciatus* (82.9%). The relative abundance of adult mosquitoes differs between districts. Adult *Cx. quinquefasciatus* was recorded across all study sites, while adult *Ae. albopictus* was found exclusively in Dolakha. In contrast, adult *Ae. aegypti*, *Ae. indicus*, *Armigeres* spp., *Anopheles subpictus,* and *Pallidus triatus* were detected only in Kaski. Moreover, the altitude did not have a statistically significant effect on the number of collected mosquitoes (GLM with Poisson distribution, p = 0.563).

### Breeding habitats associated with the presence and co-occurrence of *Ae. aegypti* and *Ae. albopictus*

Overall, about 7.6% of wet containers were infested with *Ae. albopictus*, 7.5% with *Ae. aegypti* and 6.3% contained larvae of both species, *Ae. aegypti* and *Ae. albopictus*. The location of containers (574 indoor *versus* 583 outdoor containers) significantly influenced the presence of *Ae. aegypti*, *Ae. albopictus*, and their co-occurrence, with p-values of <0.001 for all comparisons. In indoor containers, solely *Ae. aegypti* larval infestation was found in 3% and *Ae. albopictus* in 2.6%, respectively, whereas larvae of both species co-occurred in 2.4% of the inspected indoor containers. In comparision to indoor container, the odds of *Ae. aegypti* infestation were 4.47 times higher in outdoor container (95% CI: 2.60–7.70, p < 0.001), Similarly, the odds of *Ae. albopictus* presence were 5.53 times higher outdoor (95% CI: 3.02–9.42, p < 0.001). Likewise, co-occurrence of both species was significantly greater outdoor (OR = 4.50, 95% CI: 2.49–8.16, p < 0.001).

**Fig 2:**
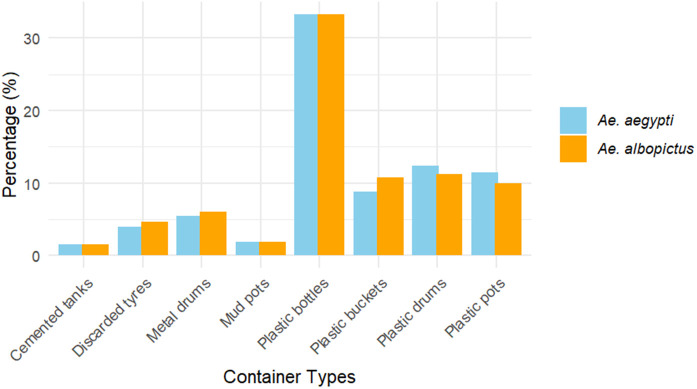
Percentage of *Ae. aegypti* and *Ae. albopictus* positive containers.

Statistical analysis showed significant associations between all wet container types (P < 0.001) for both mosquito species and their co-occurrence. Using cemented tanks as the reference group, plastic containers showed markedly higher odds of *Aedes* occurrence. The most notable findings were observed in plastic bottles, where 33.3% tested positive for both *Ae. aegypti* and *Ae. albopictus*, with an odds ratio (OR) of 33.50 (95% CI: 2.47–452.76), indicating a strong association for both species. Similarly, plastic drums showed a high prevalence, with 12.4% testing positive for *Ae. aegypti* (OR = 9.47, 95% CI: 1.28–70.09) and 11.2% for *Ae. albopictus* (OR = 8.43, 95% CI: 1.13–62.54), both with significant associations. Plastic buckets also exhibited considerable positivity, with 8.8% positive for *Ae. aegypti* (OR = 6.43, 95% CI: 0.84–49.30) and 10.8% for *Ae. albopictus* (OR = 8.1, 95% CI: 1.07–61.66). Co-occurrence of both species was found in 7.7% of plastic bucket containers (OR = 5.61, 95% CI: 0.727–43.33). On the other hand, cemented tanks, discarded tyres, metal drums, and mud pots showed lower positivity, with no significant association with *Ae. aegypti* or *Ae. albopictus* (Fig 2). Rock pools, temporary ditches, and wooden water containers had no recorded positivity for any species. The overall findings suggest that plastic containers, particularly plastic bottles and plastic drums, are strongly associated with the presence of both mosquito species. The mean ratio of *Ae. aegypti* to *Ae. albopictus* breeding preference across all container types is 1.23. This indicates that, on average, *Ae. aegypti* has a slightly higher breeding preference than *Ae. albopictus* in the observed container types (Table 2).

**Table 2:** Distribution of *Ae. aegypti* and *Ae. albopictus* larvae in different container types.

Containers type	No. of wet container	No. of *Ae. aegypti* positive container	%	BPR	% of wet containers	No. of *Ae. albopictus* positive container	%	BPR	Mean ratio *Ae. aegypti:Ae.albopictus*
**Cemented tanks**	68	1	1.4	0.2	5.8	1	1.1	0.2	1.2
**Discarded tyres**	153	6	8.9	0.6	13.2	7	7.9	0.6	1.1
**Metal drums**	166	9	13.4	0.9	14.3	10	11.3	0.8	1.1
**Mud pots**	162	3	4.4	0.3	14.00	3	3.4	0.2	**1.6**
**Plastic bottles**	6	2	2.9	5.7	0.5	2	2.2	4.4	1.3
**Plastic buckets**	194	17	**25.3**	1.5	16.7	21	**23.8**	1.4	1.0
**Plastic drums**	331	21	**31.3**	1.1	28.6	37	**42.0**	1.5	0.7
**Plastic pots**	70	8	11.9	1.9	6.0	7	7.9	1.3	**1.5**

Comparison of breeding site positivity rates, Breeding Preference Ratio (BPR), and mean ratio of *Ae. aegypti* to *Ae. albopictus* across various container types. Two maximum values for container positivity are given in bold.

Bivariate logistic regression analysis of the district-wise occurrence of *Ae. aegypti* and *Ae. albopictus* revealed significant differences across the study districts. In Chitwan, 197 wet containers were searched, with 4.6% testing positive for *Ae. aegypti* and 4.5% for *Ae. albopictus*. In Kaski, a total of 644 wet containers were examined, with 75.9% testing positive for *Ae. aegypti* (OR = 5.408, 95% CI: 1.943–15.049, p = 0.001) and 73.9% for *Ae. albopictus* (OR = 5.316, 95% CI: 1.909–14.80, p = 0.001), showing a statistically higher occurrence of both species compared to Chitwan. In Dolakha, 316 containers were searched, with 19.5% testing positive for *Ae. aegypti* (OR = 2.095, 95% CI: 0.695–6.320, p = 0.189) and 21.6% for *Ae. albopictus* (OR = 2.335, 95% CI: 0.789–7.027, p = 0.125), but neither species showed a statistically significant association. These findings highlight a stronger occurrence and association of both mosquito species in Kaski compared to Dolakha and Chitwan.

## Discussion

**Fig 3:**
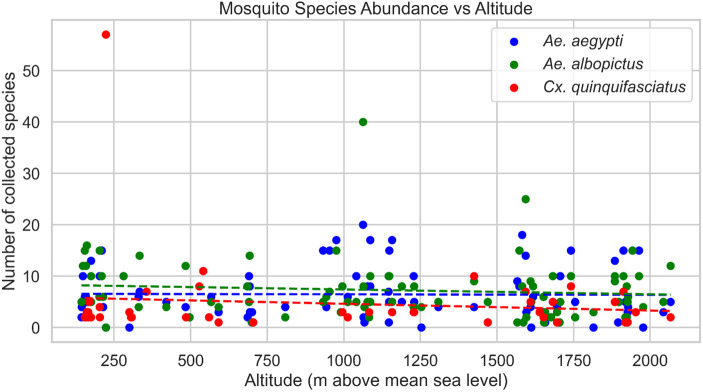
The altitudinal distribution of immature and adult life stages of predominant and medically relevant mosquitoes.

During the study period, the dengue vectors, *Ae. aegypti* and *Ae. albopictus,* and the lymphatic filariasis vector, *Cx. quinquefasciatus,* were found throughout the study transect from 140 m asl up to 2066 m asl (Fig 3). Consistent with our findings, the same vectors were found up to 2100 m asl in high mountain regions, Dhunche and Rasuwa of Nepal, and immatures of *Ae. aegypti* up to 2130 m asl in Darjeeling [28] and in Puebla, Mexico [29]. The broad distribution of these vectors may be attributed to anthropogenic factors such as habitat modification, water storage practices and climate change as well as environmental factors including rainfall patterns, seasonality, and geographical adaptability [30]. Thus, understanding these dynamics is crucial to managing the risk of mosquito-borne diseases and forecasting mosquito behavior. Our study documented a slightly higher abundance of *Ae. albopictus* than *Ae. aegypti,* which is in accordance with the studies carried out in India [31,32], Malaysia [33], Pakistan [34] and Nepal [26]. By contrast, findings from the study carried out by Tuladhar et al, 2019 [35] and Kawada et al, 2020 [36] showed the higher abundance of *Ae. aegypti.* Plastic containers, particularly drums and buckets, have been identified as the most productive breeding habitats for *Ae. aegypti* and *Ae. albopictus*, consistent with previous studies that highlight plastic containers as favorable breeding sites for these mosquito species [37,38]. The preference of *Ae. aegypti* and *Ae. albopictus* for plastic containers may be due to their natural ability to easily warm up the contained water, which stimulates the hatching of mosquito eggs and encourages larval development. This is also because long-lasting containers that retain water for longer periods, particularly artificial ones, provide ideal breeding conditions [39,40]. However, other studies reported discarded tyres as the preferred breeding habitats for *Ae. aegypti* and *Ae. albopictus* [33,41–44]. The decreased productivity of tyres in our study probably indicates their limited availability, regular disruption, direct sunlight exposure or variation in rainfall retention across ecological settings.

In contrast to our findings, natural breeding containers did not harbor *Aedes* immatures, aligning with studies conducted in Nepal and Thailand, which reported a preference for artificial breeding containers over natural ones by *Aedes* immatures [20,44]. The shift towards a preference for artificial breeding containers by *Aedes* immatures could be attributed to the ongoing evolution of the anthropophilic behavior of *Aedes* mosquitoes, strongly associated with the density of the human population and environmental factors [45]. Several factors may contribute to this preference, including container type, microclimatic conditions, and species-specific behaviors [46]. *Ae. aegypti*, typically considered a domestic mosquito, has demonstrated adaptability to outdoor breeding habitats, particularly in artificial containers such as plastic buckets, drums, and discarded tires [47,48]. *Aedes albopictus*, on the other hand, is well-documented for its ability to exploit a wide range of outdoor breeding sites, including both artificial and natural containers, making it highly adaptable to diverse environmental conditions [16]. Breeding in artificial containers close to human dwellings provides convenient access to potential hosts for blood-feeding. Both vector mosquito species breed in containers and have a close association with humans, displaying a strong preference for human blood [49,50]. Although binary logistic regression revealed significant association between container types and mosquito presence, some odds ratio showed wide confidence interval indicating low precision of estimates. This likely reflects the small number of positive containers and limited sample size within certain container categories.

Despite their habitat preferences, in areas where both species coexist, their larvae are frequently discovered together in the same breeding sites [50]. The presence of *Ae. aegypti*, *Ae. albopictus,* and their co-occurrence was significantly higher in outdoor containers, consistent with previous studies conducted in Nepal, India, and Peru [20,33,51,52]. This trend suggests that outdoor environments provide optimal conditions for mosquito breeding, likely due to greater exposure to rainfall, increased availability of water-holding containers, and minimal human intervention in emptying or treating these sites. Co-occurrence of both species in outdoor containers may increase the risk of interspecific competition, which could influence larval survival and adult emergence. Moreover, under high larval densities, *Ae. albopictus* often outcompetes *Ae. aegypti*, leading to reduced survivorship and altered adult traits, particularly in outdoor plastic containers as observed in this study [52]. However, the persistence of both species in shared breeding sites suggests that resource partitioning or differential habitat selection may play a role in mitigating competitive exclusion [50]. The presence of both species in outdoor settings is particularly concerning for vector control efforts, as it suggests increased potential for arbovirus transmission in peri-domestic environments.

In countries endemic to dengue fever, entomological indices are crucial tools for guiding vector control programs [53]. Among the commonly used entomological indices, the most widely used indices in monitoring dengue vector populations are HI and BI [54]. The Pan American Health Organization (PAHO) has divided the risk factors for dengue transmission into three levels: low (HI < 0.1%), medium (0.1% < HI < 5%), and high (HI > 5%). A House Index (HI) threshold of 1% or less or a Breteau Index (BI) threshold of five or less was considered to prevent dengue transmission [55]. In our study, three studied districts revealed HI and BI of greater than 5% indicating a high risk of dengue infection. These elevated indices imply an increased likelihood of mosquito-human contact, significantly raising the potential for a dengue outbreak. However, because sampling was conducted over a limited post-monsoon period, seasonal variation in infestation indices could not be assessed, which should be considered when interpreting transmission risk. Similar indices in other regions have been associated with dengue outbreaks, reinforcing the necessity of proactive preventive strategies in these districts [56]. Though some researchers have suggested that BI alone is not the most reliable indicator of predicting dengue transmission [54,57,58], another study demonstrated that BI outperforms other indices as the most effective predictor of dengue infections [58]. Moreover, the striking dominance of dengue vectors in both lowland and highland districts, highlights the growing threat of arboviral transmission extending beyond Nepal’s traditionally endemic low altitude zones. Furthermore, the higher infestation levels observed in Kaski are likely due to rapid urban development, extensive water storage practice and accumulation of waste linked to tourism.

In our study, altitude was found not to have a significant effect on mosquito abundance (p = 0.563). The number of mosquitoes collected did not clearly show a trend with increasing altitude, despite variations in elevations across study sites. These findings suggest that mosquito distribution may be influenced by variables other than altitude, such as local environmental conditions, habitat availability, and microclimatic elements. In contrast, it has usually been shown that mosquito populations typically decrease as elevation rises, indicating the impact of elevation on mosquito abundance [29]. Moreover, environmental variables like temperature and humidity have a significant impact on this pattern. At lower elevations, mosquitoes can breed and survive better because of the warmer temperatures and higher humidity levels [59].

The dominance of *Cx. quinquefasciatus*, along with *Ae. aegypti* and *Ae. albopictus*, indicates the species’ ability to successfully colonize and thrive across a wide range of environmental conditions and altitudes, including both lowland and highland areas. Because of its remarkable ecological adaptability, *Cx. quinquefasciatus* can thrive in a variety of settings, including both urban and rural ones. These features are highlighted by its capacity to reproduce in contaminated waters and endure in environments that are unsuitable for other species [60]. When it comes to competitive interactions, *Ae. aegypti* and *Ae. albopictus* are frequently regarded as better competitors than *Cx. quinquefasciatus*. It has been found that, *Cx. quinquefasciatus* can take advantage of habitats when conditions become limited, especially during dry seasons, whereas *Ae. albopictus* tends to dominate during wet seasons [61]. This dominance suggests their ecological adaptability, competitive advantage, and potential role as key vectors in the transmission of mosquito-borne diseases within the study region. Moreover, our study showed the absence of adult *Aedes* despite the presence of their larvae. This may be due to temporal mismatch between larval and adult sampling, low adult resting densities or limited effectiveness of traps under local environmental conditions.

Overall, our study provided comprehensive data on the abundance of two dengue vector species and the principal vector of lymphatic filariasis along three altitudinally differing districts of Nepal. The main limitation of this study is that we could not relate our study in terms of seasons and climatic determinants, which are regarded as the important factors in determining the abundance and presence of those vectors [16,20,27]. Moreover, we couldn’t link our data with the number of DF cases because the mosquito population sampling was not conducted during dengue’s peak transmission period, which could lead to insufficient data to correlate between vector species and dengue fever incidence. The co-occurrence of *Ae. aegypti* and *Ae. albopictus* in breeding habitats definitely merits further research, as it represents a complex and so far underreported ecological phenomenon in Nepal with significant implications for dengue transmission dynamics and vector control efforts.

## Supporting information

S1 Table District wise abundance of mosquito immatures. (DOCX)

S2 Table District-wise abundance of adult mosquitoes. (DOCX)

S3 Table Container type associated with presence of *Ae. aegypti, Ae. albopictus* and their co-occurrence. (DOCX)

S4 Table Bivariate logistic regression analysis for the district-wise occurrence of *Ae. aegypti* and *Ae. albopictus.* (DOCX)
