# Supplementary material for: Altitudinal and household breeding patterns of the medically important mosquitoes Aedes aegypti, Aedes albopictus and Culex quinquefasciatus in Nepal
Source: PLoS One. 2026 Mar 19;21(3):e0345285. doi: 10.1371/journal.pone.0345285 (PMC13001966; doi:10.1371/journal.pone.0345285)
Supplement: S1 Table — (DOCX) [file pone.0345285.s001.docx]

S1 Table: District wise abundance of mosquito immatures

| **Mosquito species** | **Dolakha** | | **Kaski** | | | **Chitwan** | |
| --- | --- | --- | --- | --- | --- | --- | --- |
|  | **Total collected** | **Relative abundance (%)** | **Total collected** | | **Relative abundance (%)** | **Total collected** | **Relative abundance (%)** |
| *Aedes albopictus* | 266 | 48.8 | 190 | 40.7 | | 174 | 55.9 |
| *Aedes aegypti* | 197 | 36.1 | 254 | 54.4 | | 123 | 39.5 |
| *Cx. quinquefasciatus* | 68 | 12.5 | 6 | 1.3 | | 7 | 2.3 |
| *Aedes indicus* | 5 | 0.9 | 0 | 0 | | 0 | 0 |
| *Armigeres* spp. | 4 | 0.7 | 0 | 0 | | 5 | 1.6 |
| *Anopheles subpictus* | 5 | 0.9 | 6 | 1.3 | | 0 | 0 |
| *Aedes subalbopictus* | 0 | 0 | 3 | 0.6 | | 2 | 0.6 |
| Total | 545 |  | 459 |  | | 311 |  |
