# Supplementary material for: Altitudinal and household breeding patterns of the medically important mosquitoes Aedes aegypti, Aedes albopictus and Culex quinquefasciatus in Nepal
Source: PLoS One. 2026 Mar 19;21(3):e0345285. doi: 10.1371/journal.pone.0345285 (PMC13001966; doi:10.1371/journal.pone.0345285)
Supplement: S2 Table — (DOCX) [file pone.0345285.s002.docx]

**S2 Table: District-wise abundance of adult mosquitoes**

| **Mosquito species** | **Dolakha** | | **Kaski** | | **Chitwan** | |
| --- | --- | --- | --- | --- | --- | --- |
|  | **Total collected** | **Relative abundance (%)** | **Total collected** | **Relative abundance (%)** | **Total collected** | **Relative abundance (%)** |
| *Aedes albopictus* | 3 | 33.33 |  |  |  |  |
| *Aedes aegypti* |  |  | 6 | 21.43 |  |  |
| *Culex quinquefasciatus* | 6 | 66.66 | 4 | 14.29 | 121 | 100% |
| *Aedes indicus* |  |  | 1 | 3.57 |  |  |
| *Armigeres* spp. |  |  | 2 | 7.14 |  |  |
| *Anopheles subpictus* |  |  | 14 | 50.00 |  |  |
| *Pallidus triatus* |  |  | 1 | 3.57 |  |  |
| *Aedes subalbopictus* |  |  |  |  |  |  |
| Total | 9 |  | 28 |  | 121 |  |
