# Supplementary material for: Altitudinal and household breeding patterns of the medically important mosquitoes Aedes aegypti, Aedes albopictus and Culex quinquefasciatus in Nepal
Source: PLoS One. 2026 Mar 19;21(3):e0345285. doi: 10.1371/journal.pone.0345285 (PMC13001966; doi:10.1371/journal.pone.0345285)
Supplement: S3 Table — (DOCX) [file pone.0345285.s003.docx]

S3 Table: Container type associated with presence of *Ae. aegypti, Ae. albopictus* and their co-occurrence

| **Types of wet containers** | **Number of examined containers** | ***Ae. albopictus***  **P<0.001** | | ***Ae. aegypti***  **P<0.001** | | **Cooccurrence**  **P<0.001** | |
| --- | --- | --- | --- | --- | --- | --- | --- |
|  |  | **Positive (%)** | **OR (95%CI)** | **Positive(%)** | **OR (95%CI)** | **positive (%)** | **OR (95%CI)** |
| **Cemented tanks** | 68 | 1.5 | 1 | 1.5 | 1 | 1.5 | 1 |
| **Discarded tyres** | 153 | 4.6 | 3.21(0.387-26.633) | 3.9 | 2.73(0.323-23.165) | 3.9 | 2.73(0.323-23.165) |
| **Metal drums** | 166 | 6.0 | 4.29(0.539-34.233) | 5.4 | 3.84(0.477-30.919) | 4.2 | 2.95(0.356-24.443) |
| **Mud pot** | 162 | 1.9 | 1.2 (0.129-12373) | 1.9 | 1.264(0.129-12.373) | 1.9 | 1.26 (0.129-12.373) |
| **Plastic bottles** | 6 | 33.3 | 33.50(2.479-452.769) | 33.3 | 33.5(2.479-452.762) | 33.3 | 33.5 (2.479-452.762) |
| **Plastic buckets** | 194 | 10.8 | 8.1 (1.073-61.66) | 8.8 | 6.43(0.840-49.306) | 7.7 | 5.61(0.727-43.335) |
| **plastic drums** | 331 | 11.2 | 8.43 (1.137-62.549) | 12.4 | 9.47(1.280-70.093) | 10.6 | 7.92(1.066-58.854) |
| **Plastic pots** | 70 | 10 | 7.44(0.891-62.228) | 11.4 | 8.64(1.059-71.119) | 5.7 | 4.06(0.442-37.294) |
| **Rock pools** | 3 | 0 | ND | 0 | ND | 0 | ND |
| **Temporary ditches** | 1 | 0 | ND | 0 | ND | 0 | ND |
| **Wooden water containers** | 3 | 0 | ND | 0 | ND | 0 | ND |
| **Total** | 1157 | 7.6 |  | 7.5 |  | 6.3 |  |
