# Supplementary material for: Altitudinal and household breeding patterns of the medically important mosquitoes Aedes aegypti, Aedes albopictus and Culex quinquefasciatus in Nepal
Source: PLoS One. 2026 Mar 19;21(3):e0345285. doi: 10.1371/journal.pone.0345285 (PMC13001966; doi:10.1371/journal.pone.0345285)
Supplement: S4 Table — (DOCX) [file pone.0345285.s004.docx]

**S4 Table: Bivariate logistic regression analysis for the district-wise occurrence of *Ae. aegypti* and *Ae. albopictus***

| **Districts** | **wet containers searched** | ***Ae. albopictus*** | | | ***Ae. aegypti*** | | |
| --- | --- | --- | --- | --- | --- | --- | --- |
|  |  | **Positive (%)** | **OR (95%CI)** | **P-value** | **Positive (%)** | **OR (95%CI)** | **p-value** |
| **Chitwan** | 197 | 4.5 | 1 |  | 4.6 | 1 |  |
| **Kaski** | 644 | 73.9 | 5.31 (1.909-14.80) | 0.001 | 75.9 | 5.40 (1.943-15.049) | 0.001 |
| **Dolakha** | 316 | 21.6 | 2.33(0.789-7.027) | 0.125 | 19.5 | 2.09 (0.695-6.320) | 0.189 |
